# Experimental, analytical, and simulation studies of modified concrete mix for radiation shielding in a mixed radiation field

**DOI:** 10.1038/s41598-023-44978-8

**Published:** 2023-10-17

**Authors:** Islam M. Nabil, Moamen G. El-Samrah, Ahmed Omar, A. F. Tawfic, A. F. El Sayed

**Affiliations:** 1 Physics Department, Faculty of Science, Fayoum University, Fayoum, Egypt; 2 Radiation Measurements Department, Main Chemical Laboratories, Cairo, Egypt; 3 Nuclear Engineering Department, Military Technical College, Kobry El-kobbah, Cairo, Egypt; 4 Physics Department, Faculty of Science, Cairo University, Cairo, Egypt

**Keywords:** Materials science, Physics

## Abstract

The current study assessed two concrete mixes prepared using dolomite and barite/limonite aggregates to shield against both energetic photons and neutrons. After that, a designed mix which comprised barite/goethite aggregates plus fine-powdered boron carbide additive, was proposed to improve the overall radiation shielding properties and in the same time, doesn’t compromise or even improve the physic-mechanical properties of the mature concrete. The assessment started first with intensive experimental investigations to investigate the prepared mixes’ shielding capabilities against both γ-rays and fast neutrons. Then, analytical computations were performed via number of reliable software programs such as; Phy-X, NXCom, MRCsC, JANIS-4, and MCNP5, in order to confirm the experimental results and to validate the created Monte-Carlo models. Finally, an intensive radiation shielding assessment for all concrete mixes understudy using, mainly, the validated MCNP models, was performed. The obtained results have revealed the superiority of barite mixes over the dolomite mix concerning attenuating photons moreover, the proposed designed mix has shown superiority over the other two prepared mixes considering shielding against; energetic photons, fast/thermal neutrons, and secondary emitted γ-rays, which nominates this mix to be a suitable universal shield that can be used even in mixed radiation fields.

## Introduction

In many fields, radioactive isotopes are used in essential applications, represented in industrial, agricultural, and medical applications^1–4^. The environment, animals, and people can all be harmed by the possible emitted radiation from such applications. X-rays, γ-rays, and neutrons are the most hazardous radiations considering external radiological hazards. Proper shielding material is well recognized for providing an effective barrier between the radioactive source and the surrounding area where residents or workers are, which is essential to decrease the exposure thus, the received dose^5^. Based on the former, the importance of continuous development regarding radiation shielding materials has increased.

Many researchers are interested in discovering better materials to use as promised radiation shielding materials besides having other physic-mechanical properties suitable to the intended application^6–8^. It is generally accomplished by using composite shields, which allow the use of versatile different materials all included in one matrix, such as dense materials like lead or tungsten, which are effective in attenuating energetic photons, light elements like hydrogenous compounds due to their moderation and removal capabilities against fast neutrons, and elements or compounds which have considerable thermal neutron absorption affinity like boron, lithium, and their suitable compounds^9–12^.

By speaking on concrete, considering being the most widely employed construction material around the world, plenty of researchers have worked on modifying the mix design to attain reliable radiation shielding concrete (RSC) with proper physical and mechanical properties, which are essential, especially that the designed (RSC) usually utilized in nuclear installations and should carry and resist static and dynamic loads^13,14^. Moreover, when the designed (RSC) is intended to be used in certain nuclear and radiological applications that encompass the emission of various hazardous radiations, such as storage of spent nuclear fuel or high-level radioactive wastes, the selected ingredients should consider all these kinds of radiations effectively and simultaneously without ignoring any possible secondary radiation that may arise^15–18^.

Most of the studies have discussed the feasibility of using alternative coarse aggregates as full replacements or as a part of the conventional ones, such as using magnetite, hematite, ilmenite, steel slag, galena, serpentine, tourmaline, and so on^19–22^. Several researchers have also discussed using some specific ground or fine powdered materials, either natural or synthetic, as a portion of the fine aggregates or as additives such as bentonite, ground heavy glass, fine powdered borosilicate glass, powdered colemanite, etc.^23–27^. Most of the former studies focused on improving the shielding capabilities of the proposed concrete mixes either against energetic photons or against neutrons and sometimes against both photons and fast neutrons without considering dealing with the thermalized neutrons in a proper way. However, when absorption of thermal neutrons needs to be considered, adding “neutron poisons” is essential, especially cheap and available boron compounds. The problem that we have tried to solve in the current study is that most of the studied boron compounds which can be added to concrete to raise the thermal neutrons absorption capability of the prepared concrete mix and, at the same time, reduce the emitted secondary γ-rays, is usually harmful to the cement hydration reaction, microstructure, and/or the other mechanical properties as proved by many studies^8^. Proposing adding very fine powdered commercial boron carbide has been introduced in the current study after experimentally proving its improving effect upon the cement hydration reaction, microstructure, and the compressive strength of the attributed cementitious composites, unlike the case with the former studies, which tried to add boron compounds to concrete but, unfortunately, that led to harmful consequences^22,28^.

The radiation shielding assessment is usually performed employing numerous systems for experimental measurements considering gamma rays and neutrons transmission experiments to derive some important characteristic shielding parameters such as linear attenuation coefficient (μ), macroscopic fast neutrons removal cross-section (Σ_R_), and thermal/epithermal neutrons absorption cross section (Σ_Abs_)^19,21,29–31^. Also, a considerable number of reliable theoretical studies has included analytical radiation shielding investigations, which are performed via various software programs that rely on validated analytical / semi-empirical models, cross-sectional databases, and/or rigid simulation engines to compute the needed shielding parameters such as; Phy-X/PSD^32–34^, FLUKA^35,36^, WinXcom^37^, NXCom^38^, MRCsC^39^, MCNP^40,41^, and GEANT4^42^, which is an effective way for the assessment during the initial design phase of the shield or when the required tools for the experimental measurement are unavailable.

During this study, two prepared concrete mixtures; one of them comprised dolomite as coarse aggregates and represented a traditional concrete mix (DoC), and the other one comprised barite ore as coarse aggregates and a balanced mixture of limonite/sand as fine aggregates (BLC), were assessed experimentally and analytically to examine their shielding capabilities against γ-rays and neutrons and, in the same time, to validate created Monte-Carlo models to expand the study reliably. After that, a third concrete mix was proposed and designed to reveal proper shielding efficiency against all possible types of hazardous external radiations while not compromising or improving the physic-mechanical properties of the produced concrete. The third proposed mix comprises barite coarse aggregates, goethite/sand fine aggregates, and fine boron carbide additive.

The aggregates incorporated in the current study, dolomite, barite, limonite, and goethite, are feasible to be employed in concrete manufacturing as they are readily available in their mineral forms around the world and in low costs because they are all utilized in their mineral forms without further processing^43^. Moreover, the “materials and methods” section provides more details considering these aggregates.

The corresponding shielding parameters of the proposed mix have been predicted by the validated Monte-Carlo models created by the simulation code, MCNP5^40^, and compared with the other obtained parameters for the other two prepared mixes to examine if this designed concrete mix can be used as a universal shield which is capable of facing different types of hazardous external radiations or not.

## Materials and methods

### Materials and mix design

Through this study, two concrete mixes were prepared and investigated experimentally and analytically to assess their radiation shielding capabilities against both γ-rays and neutrons. Based on the results that will be discussed in detail in the coming sections, a newly designed mix has been proposed and analytically studied to assess its competency as a universal shield that can be utilized in a field of mixed radiation, considering its shielding efficiency against secondary radiation.

Barite [BaSO_4_] and dolomite [CaMg (CO_3_)_2_], which were obtained from El-Bahariya Oasis, Western Desert, Egypt, were used as coarse aggregates for both BLC and DoC prepared mixes, respectively. The fine aggregates for both mixes are limonite/sand balanced mixture and silica sand, respectively. The binder for both mixes is Portland blast furnace slag cement (Labeled PBFSC) CEM/B-S 42.5 N, which is compatible with ASTM C150^44^ considering adding 10% “by replacement” silica fume (SF) in case of BLC only with total cementing material content of 500 kg per concrete cubic meter, the volumetric ratio between coarse and fine aggregates is 2:1, and the water to cement (w/c) ratio was chosen to be 0.5. Coarse aggregates were sieved and selected to be in the 5–20 mm range, and fine aggregates with a particle size range of 0.3–5 mm.

The proposed mix consists of barite coarse aggregates and a balanced mixture of goethite/silica sand as fine aggregates. Goethite [α-FeO(OH)], Like the other aggregates, obtained from El-Bahariya Oasis, Western Desert, Egypt. Goethite has been chosen for the proposed mix as an alternative for limonite as it possesses physic-mechanical properties better than that for limonite such as density which is 4.0 g/cm^3^ compared to 2.26 for limonite and water absorption value which is reasonably lower than that for limonite (13% compared to about 33% for the used limonite). Finely powdered boron carbide [C_1.7_B_13.3_], from Feldco International Company, Ladera Ranch, California, USA, with an average particle size of 2.6 μm and 98% purity, has been considered to be added as a replacement for the fine aggregates’ content with a 2.5 wt% of the overall concrete mix weight. The selected fine powdered boron carbide was found to be beneficial to the physic-mechanical properties of concrete as it enhances the cement hydration reaction due to the filler effect; thus, the mechanical properties, especially the compressive strength of the produced concrete, besides increasing the workability time via retarding the setting time; however, the latter should be adjusted carefully to avoid any possible segregation^25^. The selected w/c ratio for this proposed mix is 0.4, considering adding 2% Sikament-NN superplasticizer (type G) to enhance the overall workability. The cementing materials are the same as those used for the formerly prepared mixes. For a better understanding, the chemical compositions of the used materials and the mixes’ proportions are presented in Tables 1 and 2, respectively.

**Table 1:** Chemical compositions and densities of the used cementing materials and aggregates.

Oxide	Chemical compositions (wt%)						
	PBFSC	SF	Barite	Dolomite	Limonite	Goethite	Sand
CaO	57.07	0.160	1.590	37.90	4.160	6.111	0.521
SiO_2_	23.33	96.81	1.160	2.240	16.30	11.08	95.84
Al_2_O_3_	5.910	0.250	0.640	0.950	2.970	3.051	2.210
Fe_2_O_3_	3.290	0.450	20.84	0.610	68.10	62.30	0.820
MgO	3.100	0.260	1.630	15.03	0.650	0.893	0.101
MnO	–	0.050	1.100	–	–	0.263	–
SO_3_	2.900	0.140	4.420	0.390	2.900	1.710	0.110
K_2_O	0.250	0.280	0.340	0.070	0.740	1.620	0.690
Na_2_O	0.240	0.140	–	0.250	0.990	1.314	0.270
TiO_2_	0.080	–	–	0.130	1.290	1.341	0.120
BaO	–	–	67.10	–	–	–	–
Cr_2_O_3_	–	–	0.170	–	0.850	0.416	–
LOI	2.970	0.980	0.200	42.43	1.100	9.500	0.210
Density,g/cm^3^	3.15	2.26	4.40	2.69	2.28	4.00	2.68

**Table 2:** Mix proportions for the studied concrete mixes.

Mix	Concrete proportions (kg/m^3^)							
	Cement	Fine aggregates		Coarse aggregates			Pozzolans/additives	
	PBFSC	Sand	Limonite	Goethite	Barite	Dolomite	SF	C_1.7_B_13.3_
BLC	450	270	226	–	1798	–	50	–
DoC	500	555	–	–	–	1126	–	–
BGC	450	380	–	236	1892	–	50	81

The absolute volume method was used to prepare the concrete mixtures, as recommended by the American Concrete Institute (ACI)^45^. Water absorption during mixing was avoided using only aggregates in their saturated surface dry (SSD) form^28,46^. Cubic and cylindrical molds were used to cast concrete samples, which were consolidated using a vibrating table and left to set for 24 h before demolding. Specimens were then submerged in curing water tanks before the testing date.

### γ-rays shielding assessment

#### Experimental measurements

For this investigation, cylindrical samples were cast for each concrete mix and then sliced to varying depths. Each sample measured 20 cm in length and 10 cm in diameter. The gamma-ray energies employed during these attenuation tests are 0.081, 0.356, 0.662, 1.173, and 1.332 MeV for Ba-133, Cs-137, and Co-60, respectively^47^. Using a NaI(Tl) scintillation detector coupled with a multichannel analyzer running software (UCS-30) version 1.1.06 USB, Spectrum Technique 2010, the gamma rays emanating from the sources and those uncollided beams after the sample thicknesses were measured.

**Figure 1:**
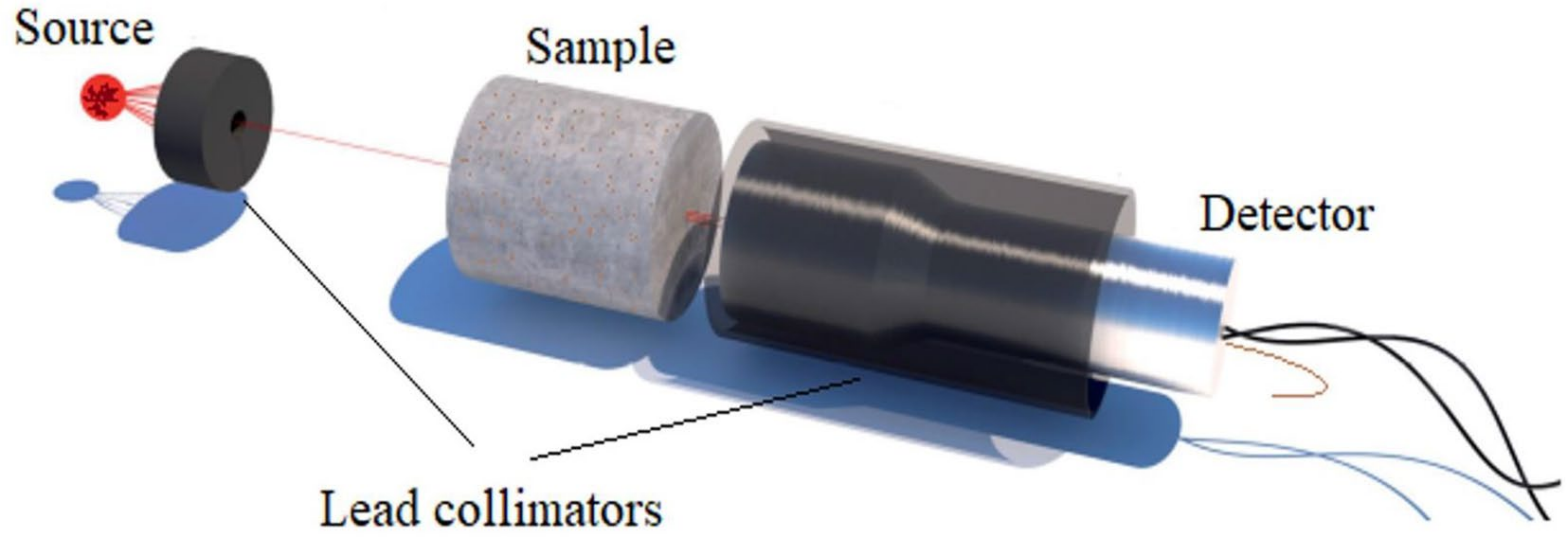
The experimental setup for the gamma rays attenuation measurements.

The gamma radioactive source was contained in a 3 cm lead holder (source collimator) with a 3 mm diameter aperture, and the scintillation detector was shielded by lead blocks (detector collimator) to eliminate the effects of scattered gamma rays and background radiation and ensure accurate readings. The collimators used along with a straight alignment for the components of the experimental setup were mainly to ensure narrow beam geometry to ignore the build-up factors and to get accurate characteristic shielding parameters for the mixes understudy^13,48,49^. All the measurements were taken as triplets. As shown in Fig. 1, the gamma-ray beam, the samples, and the detector were all set on a horizontal plane with a pre-fixed distance of 31 cm between the source and the face of the detector. The transmission curves have been obtained by measuring the intensity of the uncollided γ-ray passed through the material at several thicknesses and applying the Beer–Lambert Equation. Then, the attenuation coefficient at the studied energy for each sample could be calculated based on the following equation^4,46^.

1 $$I\left( \gamma \right) \, = \, I_{o} e^{ - \mu \, x}$$

where* I* is the intensity of transmitted gamma photons through the material, *I*_*o*_ is the intensity of the primary γ-rays emitted from the source,* x* is the attenuator thickness, and *μ* is the linear attenuation coefficient. The linear attenuation coefficient *μ* is necessary to calculate the thicknesses needed to reduce the primary radiation to half, *HVL*, or tenth, *TVL*, of its original value and to get the required shield thickness to reach safe doses^4,46^. The following equations can be used to calculate both the half-value layer (*HVL*) and tenth-value layer (*TVL*)^36^:

2 $$HVL = \frac{ln2}{\mu }$$

3 $$TVL = \frac{ln10}{\mu }$$

#### Analytical methods

##### Phy-X/PSD software

Phy-X/PSD software (Phy-X) is an online program that computes numerous parameters related to the shielding and attenuation capability of the studied material, dosimetry, etc.^32^. Many calculations can be performed using the Phy-X input file, including those for effective atomic number (*Z*_*eff*_), mass attenuation coefficients (*µ*_*m*_), linear attenuation coefficients (*µ*), half value layer (*HVL*), etc. The elemental composition, density, and energy range in MeV were predefined as input parameters. Also, the desired parameters were selected as the output data to investigate and assess the studied samples. The obtained results from Phy-X were then compared to the experimentally measured values and the results obtained from MCNP5 to assess the agreement among the three employed methods.

##### MCNP gamma transport simulation code

**Figure 2:**
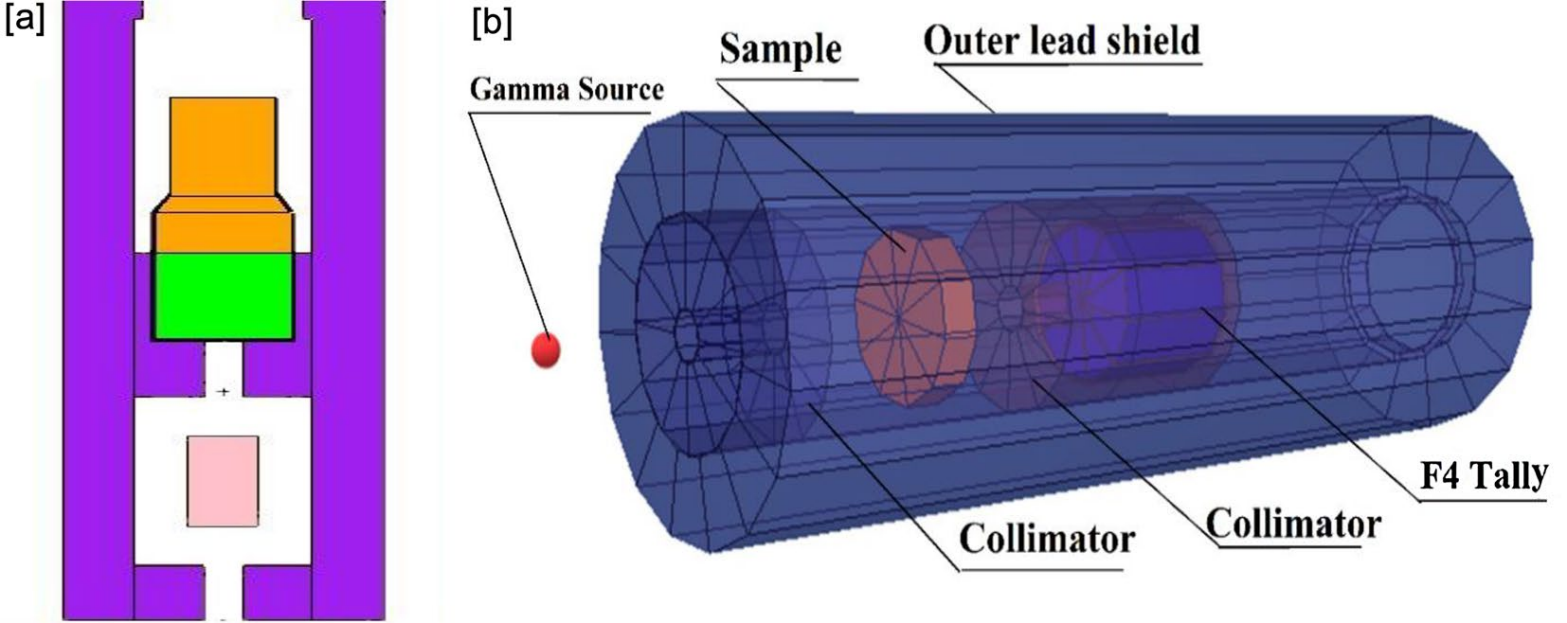
(**a**) 2-D view and (**b**) 3-D dynamic view of the Gamma rays’ attenuation simulation system.

The simulations of gamma rays irradiation of the investigated concrete mixes were performed using Monte-Carlo code (MCNP5)^40^ with mono-energetic point sources representing γ-energy lines of 0.081, 0.356, 0.6616, 1.173, and 1.3325 MeV for the radioactive sources B-133, Cs-137, and Co-60 respectively. The code simulates the transit of gamma photons while considering the physical interaction principles [photoelectric (PE), compton scattering (CS), and pair production processes (PP)]^40^. MCNP simulation code is supported by the ENDF/B-VII nuclear database, which is utilized to estimate the mass attenuation coefficients (µ_m_, cm^2^/g) of the investigated samples^40^. MCNP5 input files need precise predefined data (e.g., source dimensions, source-to-detector distance/alignment, geometry, elemental composition, etc.), as seen in Figs. 2a,b. All predefined parameters have been considered to imitate the experimental system. Six components, radioactive source, primary γ-rays collimator, sample, secondary γ-rays/detector collimator, and detector, were described by a cell in the text format file. The radioactive source was positioned inside the back of a lead collimator of the primary γ-rays^50^. A γ-rays point source was identified as an SDEF mono-energetic beam for each input file^40,51^. The samples were created as a layer positioned between the source and the detector. In addition, the elemental composition and densities of the studied samples were created in the material card of the text file. The detector was configured inside a lead collimator considering the secondary γ-rays. The command tally F4:P was selected to determine the average track length of the incident γ-rays emitted from simulated γ-source. The created (detector, source, collimators, and samples) were surrounded by an outer lead shield. All the calculations are carried out on a core i5–2.3 GHz processor with several histories of NPS (10^7^) for each file to achieve random statistical errors of better than (1%) and took about 12 min/run for a total of (12) input files.

### Neutrons shielding assessment

#### Experimental measurements

**Figure 3:**
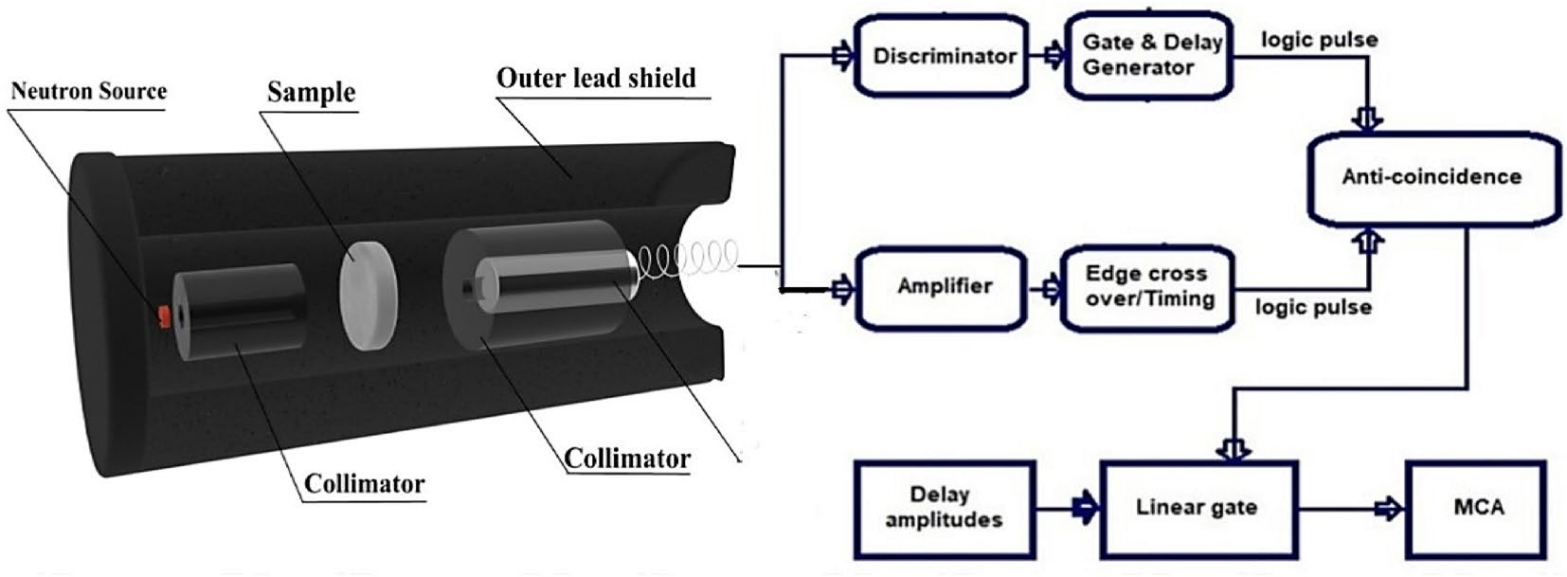
Block diagram and experimental setup of neutrons measurement system.

A collimated beam of fast neutrons emitted from a Cf-252 radioactive source was detected using a stilbene (4 × 4 cm crystal dimensions) organic scintillation detector coupled with a photomultiplier tube. The activity of ^252^Cf was measured to be 1.05 mCi at the time of the measurements. Fast neutron fluxes transmitted through different thicknesses of the concrete mixes under study were used to calculate the corresponding effective macroscopic fast neutrons removal cross-section^52^. Table. 3 shows the ^252^Cf source radiological characteristics. When neutrons and γ-rays engage with the stilbene crystal, the produced recoiling protons and electrons produce respective pulses, which can be distinguished using the pulse shape discrimination technique. The block diagram and experimental setup are depicted in Fig. 3. Macroscopic fast neutrons removal cross-section (*Σ*_*R*_) was determined from the slope of the attenuation curves, then the corresponding half value layer (*HVL*_*fn*_) and relaxation length (*λ*_*fn*_), which can be defined as the average distance that can be traveled by a fast neutron before making notable interaction with the medium, for each concrete mix was obtained by using the following equations^4,46,53^:

4 $$I_{x} = I_{0} e^{{ - \Sigma_{R} x}}$$

5 $$HVL_{fn} = \frac{ln2}{{\Sigma_{R} }}$$

6 $$\lambda_{fn} = \frac{1}{{\Sigma_{R} }}$$

**Table 3:** Some important radiological properties of the used ^252^Cf source.

Property	Value
Alpha particle energy	6.12 MeV
Effective half-life	2.65 years
Alpha decay half-life	2.73 years
Spontaneous fission half-life	85.5 years
Initial activity, when produced	100 mCi
Average neutron energy	2.35 MeV
Neutron emission rate	1.721 × 107 n/sec
Fission rate	6.2 × 105 f./sec. μgm

*I*_*x*_ is the intensity of transmitted neutrons through the material, *I*_*o*_ is the initial neutronic intensity detected by the spectrometer without any absorber, *and x* is the attenuator thickness.

#### Analytical methods

##### MRCsC and NXCom software programs

The sample’s shielding effectiveness against fast neutrons was assessed first by computing the effective macroscopic fast neutrons removal cross-section (*Σ*_*R*_) employing the software programs MRCsC^39^ and NXCom^38^. Both programs have a built-in database of microscopic mass removal cross-sections. $\left({\Sigma }_{R}/\rho \right)$ for most of the naturally occurring elements and both compute (*Σ*_*R*_) using the mixture rule as follows^4,46^;

7 $$\Sigma_{R} = \mathop \sum \limits_{1}^{n} \rho_{s} w_{i} \left( {\Sigma_{R} /\rho } \right)_{i} = { }\mathop \sum \limits_{1}^{n} \rho_{i} \left( {\Sigma_{R} /\rho } \right)_{i}$$

where ${\rho }_{s}$ and ${\rho }_{i}$ are the shield density (g/cm^3^) and the density of the ith element, respectively.

Nevertheless, the built-in database in MRCsC is the most updated considering the latest version of the Evaluated Nuclear Data Library, “ENDF/B-VIII,” published in 2018^54^. As a very important observation, the MRCsC program was found to overestimate the parameter’s value slightly, but the NXCom program has shown slight underestimation while calculating the parameter; thus, taking the mean value of the results obtained by both programs was found in a good agreement with the corresponding accurate experimentally obtained parameter.

Secondly, based on the obtained average value of (*Σ*_*R*_), half value layer (*HVL*_*fn*_), in cm, which is the needed thickness of the material under study to remove 50% of the incident fast neutrons, and the relaxation length (*λ*_*fn*_), in cm, that can be well-defined as the average distance that a fast neutron can travel before making notable interaction with the medium, were then calculated based on the abovementioned equations, Eqs. (5 and 6)^4,46^:

##### MCNP neutronic simulation

**Figure 4:**
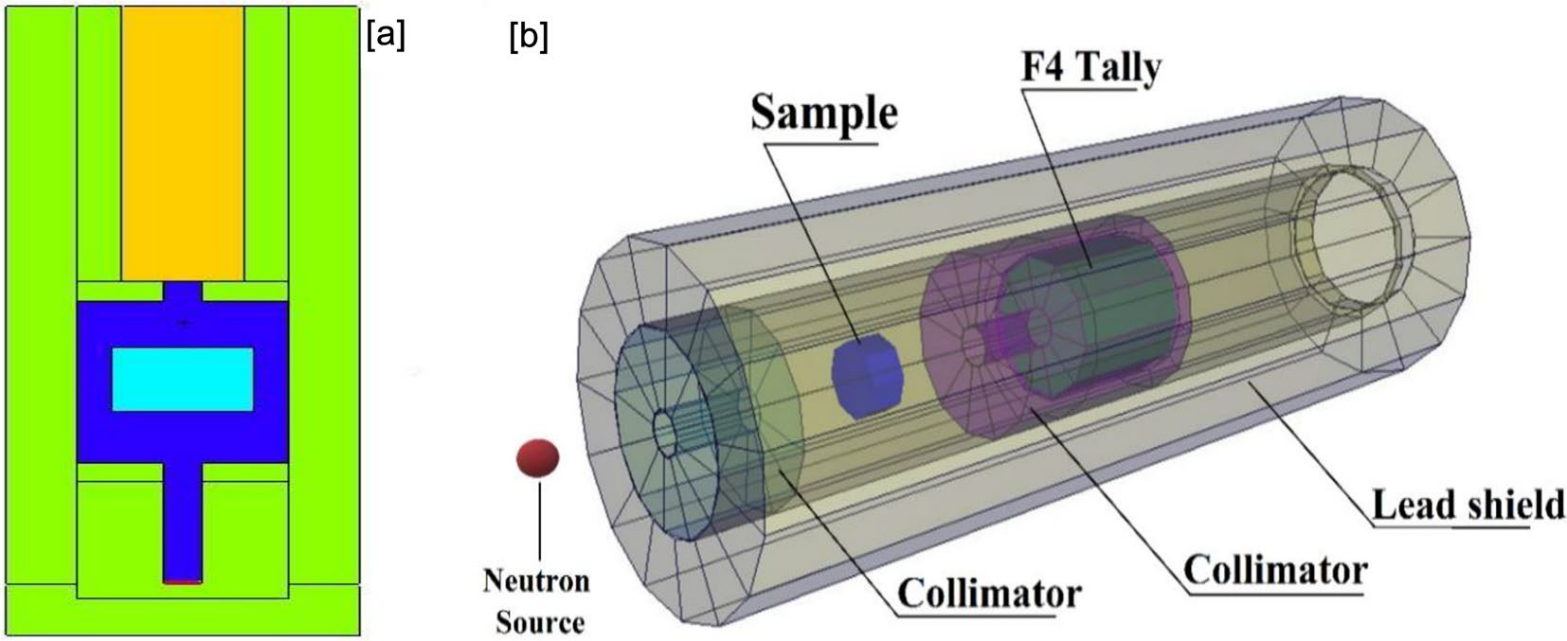
(**a**) 2-D view and (**b**) 3-D dynamic view of the neutrons’ attenuation simulation system.

The simulations of neutron irradiation of the investigated samples were performed using MCNP5 to calculate the parameters taken into account by the experimental measurements. The same components were created as the gamma radiation system discussed, but there was a little critical difference, as seen in Figs. 4a,b. The neutron source was described as a californium point source (Cf-252) with a Watt fission spectrum covering the energy range 1–10 MeV to consider fast neutrons and to compute the corresponding removal cross-section^21,40,55^. The command tally F4:N determines the average track length of the incident neutrons emitted from the simulated neutron source. The Mode Card N and P were used to emphasize the detection of neutrons and secondary gamma simultaneously in the detector cell. The created (detector, source, collimators, and samples) were surrounded by an outer lead shield. All the calculations are carried out with several histories of NPS (10^7^) for each file to achieve random statistical errors of lower than (1%) and took about 10 min/run for a total of (6) input files.

### Conclusive γ-rays and neutrons shielding assessment using MCNP-5 Code

After validating the created MCNP models based on the methodology mentioned above, the prepared mixes, BLC and DoC, besides the third proposed mix, BGC, will all be investigated in detail, considering their attenuation capabilities against energetic photons, fast neutrons, and thermal neutrons.

The abovementioned MCNP models, to be validated, will be used to assess γ–rays and neutrons shielding capabilities of the three concrete mixes and to assess the possible secondary reactions that may arise from radiation/material interaction, especially when the absorber constituents absorb neutrons.

In addition to the former, the mix’s capability to absorb thermalized neutrons was investigated by computing the macroscopic thermal neutrons absorption cross-section (*Σ*_*abs*_) using JANIS-4 software^56^ to provide better explanation while considering the results to be obtained from the MCNP simulated neutronic model. JANIS-4 is an improved NEA Java-based Nuclear Data Information System version that ensures direct access to evaluated nuclear-certified libraries such as CINDA, EAF, EXFOR, and ENDF^56^. ENDF/B-VIII library, which is the most updated version of the ENDF database, was chosen for the calculations considering E_n_ = 0.025 eV^54^, and the parameter was computed employing the following equations^4,16^;

8 $$\left( {\Sigma_{abs} /\rho } \right)_{i} = \frac{N}{{\rho_{i} }}\left( {\sigma_{a} } \right)_{i}$$

9 $$N = \frac{{\rho_{i} N_{A} }}{{M_{i} }}$$

10 $$\Sigma_{abs} \left( {E_{n} { } = { }0.025\,{\text {eV}}} \right) = \mathop \sum \limits_{1}^{n} \rho_{s} w_{i} \left( {\Sigma_{abs} /\rho } \right)_{i}$$

where; ${({\sigma }_{a})}_{i}$, ${M}_{i}$,${\left({\Sigma }_{abs}/\rho \right)}_{i}$, *N*_*A*_ and *N* are the microscopic thermal neutron absorption cross-section (cm^2^/atom), the molar mass (g/mol), the macroscopic thermal neutron mass absorption cross-section (cm^2^/g), Avogadro’s number (atom/mol), and the atomic density of the element (atom/cm^3^), respectively.

The main aim of this phase is to investigate the functionality of the proposed mix, especially when placed in a mixed radiation field, and to examine the possible secondary nuclear reactions, such as (n, α) and (n, γ) reactions, upon reacting of neutrons with the shield constituents.

## Results and discussion

### Gamma rays and neutrons shielding assessment of the prepared mixes

**Figure 5:**
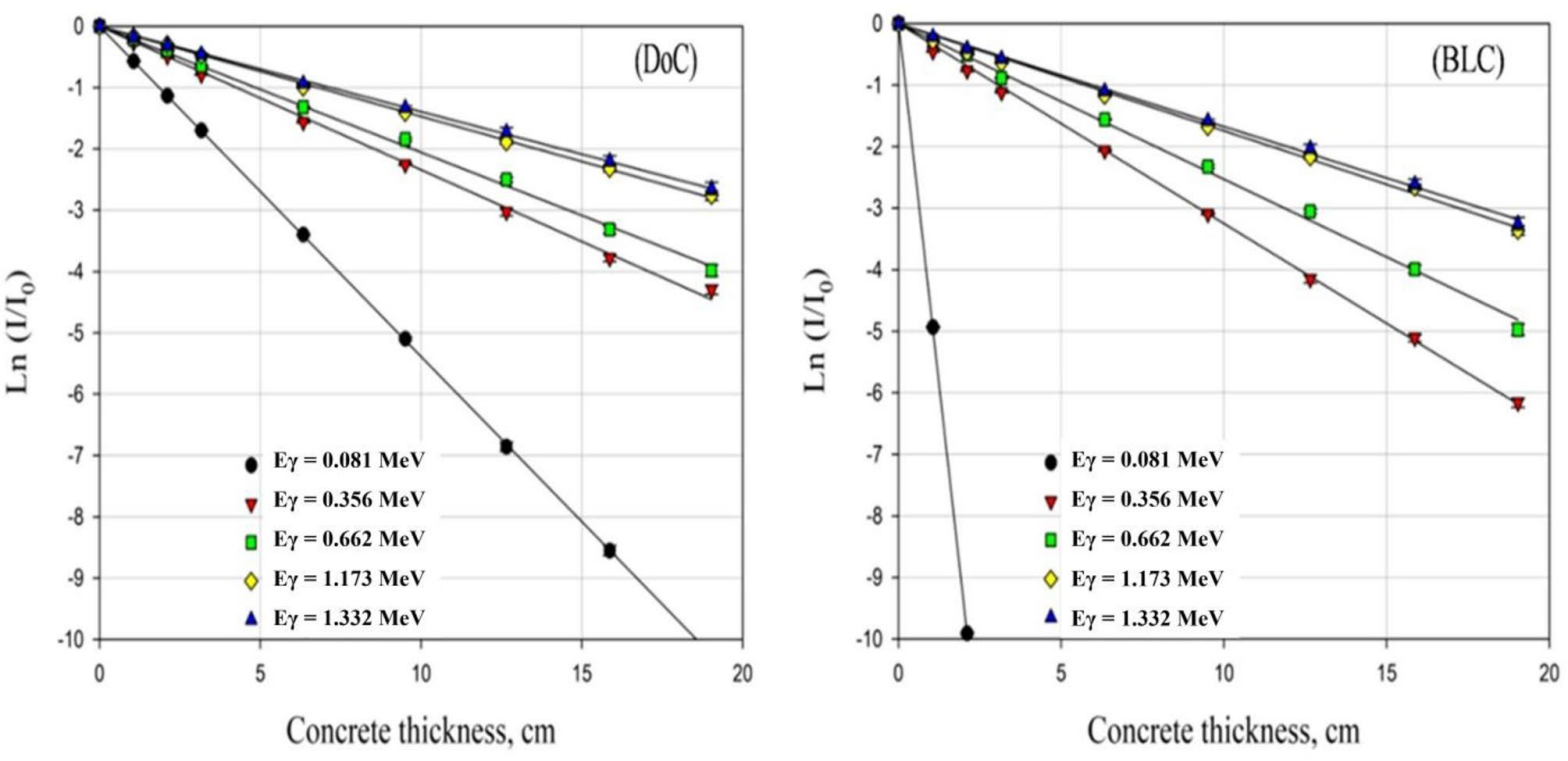
The experimentally obtained transmission curves of γ-rays relative intensity versus the thickness of the studied concrete mixes.

Based on the transmission curves presented in Fig. 5, the absolute value of the derived slope has been taken as the linear attenuation coefficient value at the corresponding photons’ energy.

As mentioned before, the attenuation capabilities of the two prepared concrete mixes against gamma rays have been assessed at five gamma energies using three radioactive isotopes as a trial to cover three different energy regions: the low, intermediate, and high energy regions.

Linear attenuation coefficients were obtained experimentally and analytically, as described above. The calculated percent difference between the experimentally obtained values and the simulated ones using MCNP5 and also those between the experimental values and the calculated ones obtained by the Phy-X program indicate great agreement and compliance, almost up to 95% agreement at most of the studied energies, which validates the created MCNP model, as presented in Table 4.

**Table 4:** The measured and computed linear attenuation coefficient (*µ*) values obtained experimentally and from MCNP5 and Phy-X for DoC and BLC mixes.

Energy, (MeV)	linear attenuation coefficient (*µ*) with a percent difference									
	DoC					BLC				
	EXP	MCNP5	%Dif	Phy-X	%Dif	EXP	MCNP5	%Dif	Phy-X	%Dif
0.081	0.537	0.523	2.61	0.537	0.02	4.704	4.779	1.59	4.811	2.28
0.356	0.233	0.246	5.58	0.248	6.44	0.338	0.345	2.07	0.359	6.21
0.661	0.199	0.189	5.03	0.190	4.43	0.256	0.242	5.47	0.236	7.68
1.173	0.146	0.144	1.37	0.145	0.97	0.177	0.17	3.95	0.172	2.62
1.332	0.139	0.135	2.88	0.135	2.55	0.17	0.159	6.47	0.161	5.22

Based on the obtained Linear attenuation coefficients at the studied five energies, half value layer and mean free path values at the corresponding energies were all calculated to compare the prepared mixes and assess their shielding efficiencies, as shown in Table 5.

**Table. 5:** Gamma-ray attenuation parameters (µ, HVL, and TVL) were obtained experimentally, MCNP5 and Phy-X for DoC and BLC mix.

Eγ, (MeV)	Gamma-rays attenuation parameters																	
	DoC									BLC								
	*µ* (cm^−1^)			HVL (cm)			TVL (cm)			*µ* (cm^−1^)			HVL (cm)			TVL (cm)		
	MCNP	EXP	Phy-X	MCNP	EXP	Phy-X	MCNP	EXP	Phy-X	MCNP	EXP	Phy-X	MCNP	EXP	Phy-X	MCNP	EXP	Phy-X
0.081	0.523	0.537	0.537	1.325	1.291	1.291	4.403	4.288	4.288	4.779	4.704	4.811	0.145	0.147	0.144	0.482	0.489	0.479
0.356	0.246	0.233	0.248	2.818	2.975	2.795	9.360	9.882	9.285	0.345	0.338	0.359	2.009	2.051	1.931	6.674	6.812	6.414
0.661	0.189	0.199	0.19	3.667	3.483	3.648	12.183	11.57	12.12	0.242	0.256	0.236	2.864	2.708	2.937	9.515	8.994	9.757
1.173	0.144	0.146	0.145	4.814	4.748	4.780	15.990	15.77	15.88	0.17	0.177	0.172	4.077	3.916	4.030	13.545	13.01	13.38
1.332	0.135	0.139	0.135	5.134	4.987	5.134	17.056	16.56	17.06	0.159	0.17	0.161	4.359	4.077	4.305	14.482	13.54	14.30

The superiority of BLC mix over DoC mix has been observed at all studied energies, which is logical due to the employment of barite as coarse aggregates in the BLC mix in addition to the use of limonite, which is a hydrous iron ore as a portion of the used fine aggregates in this concrete mix. Using the former ores or minerals that contain considerable high-Z elements increased the overall high-Z elements content, especially barium (Ba-56) and iron (Fe-26), in the ultimate concrete mix, which, in turn, increased the effective atomic number of the mix and improved the attention capability against energetic photons.

The results show that μ values at the studied energies decrease as the incident photons’ energy increases; thus, HVL and MFP values increase, which is attributed to the fact that the photon escaping probability in the attenuating medium increases with its energy and thus, the attenuation capability of the shield decreases^4,6^.

The superiority of the BLC mix over the DoC mix at low energies was found to be tremendous as the photoelectric interaction mechanism, which is proportional to Z^4.5^, is the dominant interaction mechanism in the low energy range. This superiority decreases notably, while still existing, in the intermediate energy range due to the dominancy of the Compton scattering mechanism, which is the least Z-dependent interaction mechanism among the main gamma rays interaction mechanisms with the matter^4,6^.

**Figure 6:**
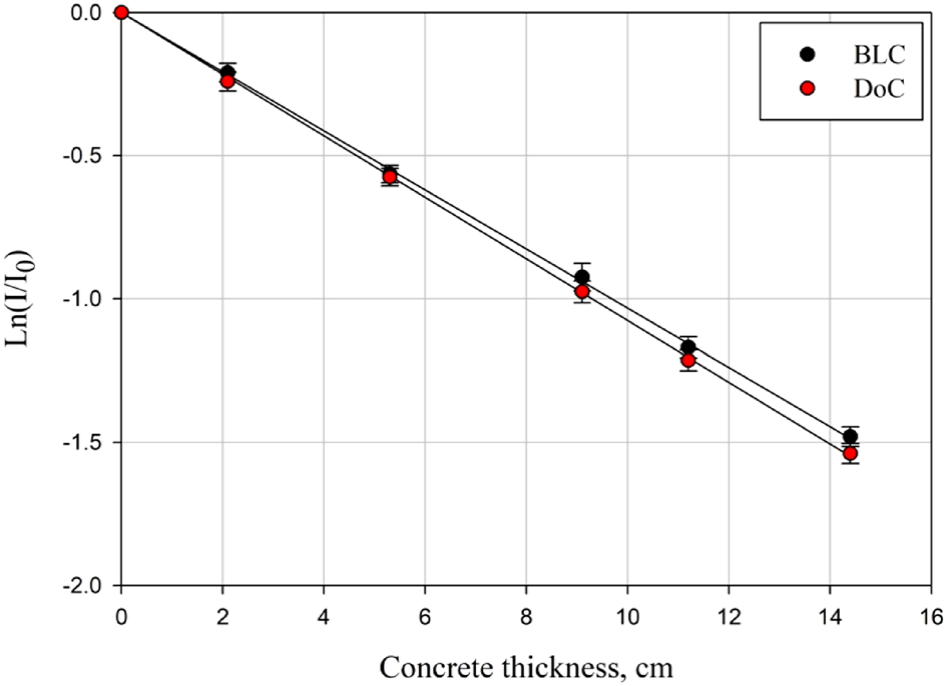
The experimentally obtained transmission curves of the incident energetic neutrons versus the thickness of the studied concrete mixes.

Based on the integrated neutronic transmission curves presented in Fig. 6, the derived slope's absolute value represents the effective macroscopic fast neutrons removal cross-section, *Σ*_*R*_, value of the studied concrete mix.

The calculated percent differences between the experimentally obtained macroscopic fast neutrons removal cross-sections and the simulated ones using MCNP5 and also those between the experimental values and the calculated ones obtained as the mean values of NXCom and MRCsC programs show significant compliance, above 98% agreement considering MCNP5 model results, which ensures the model validation, and above 95% agreement considering NXCom and MRCsC programs obtained results in comparison to the experimentally obtained values as shown in Table 6.

**Table 6:** Macroscopic fast neutrons removal cross-section (Σ_R_), which was obtained experimentally and by using MCNP5, MRCsC, and NXCom for DoC and BLC mixes along with the corresponding percent difference.

Parameter	DoC					BLC				
	EXP	MCNP5	%Dif	Avg. M&N	%Dif	EXP	MCNP5	%Dif	Avg. M&N	%Dif
Σ_R,_ (cm^−1^)	0.108	0.107	0.931	0.107	0.931	0.103	0.101	1.961	0.099	3.960

Despite having higher density, the BLC mix has a lower capability to shield against fast neutrons than the DoC mix, as denoted by the computed fast neutrons shielding parameters in Table 7. The reason is that despite having considerable content of heavy elements, the BLC mix's content of light elements, which is the most efficient in moderating and thermalizing fast neutrons via elastic scattering, is lower than that of the DoC mix. The former clarifies the importance of the best use of concrete as a matrix suitable for containing versatile and different elements to ensure adequate synchronized shielding against different types of radiation.

**Table 7:** Fast neutrons attenuation parameters (Σ_R_, HVL, and λ) obtained experimentally and from MRCsC and NXCom, Avg. (M&N), for DoC and BLC mixes.

Fast neutrons attenuation parameters																	
DoC									BLC								
Σ_R_ (cm^−1^)			HVL (cm)			λ (cm)			Σ_R_ (cm^−1^)			HVL (cm)			λ (cm)		
MCNP5	EXP	Avg. M&N	MCNP5	EXP	Avg. M&N	MCNP5	EXP	Avg. M&N	MCNP5	EXP	Avg. M&N	MCNP5	EXP	Avg. (M&N)	MCNP5	EXP	Avg. M&N
0.107	0.108	0.107	6.478	6.418	6.730	9.346	9.259	9.709	0.101	0.103	0.099	6.863	6.729	7.001	9.901	9.708	10.11

### Conclusive γ-rays and neutrons shielding assessment for the three concrete mixes understudy

**Figure 7:**
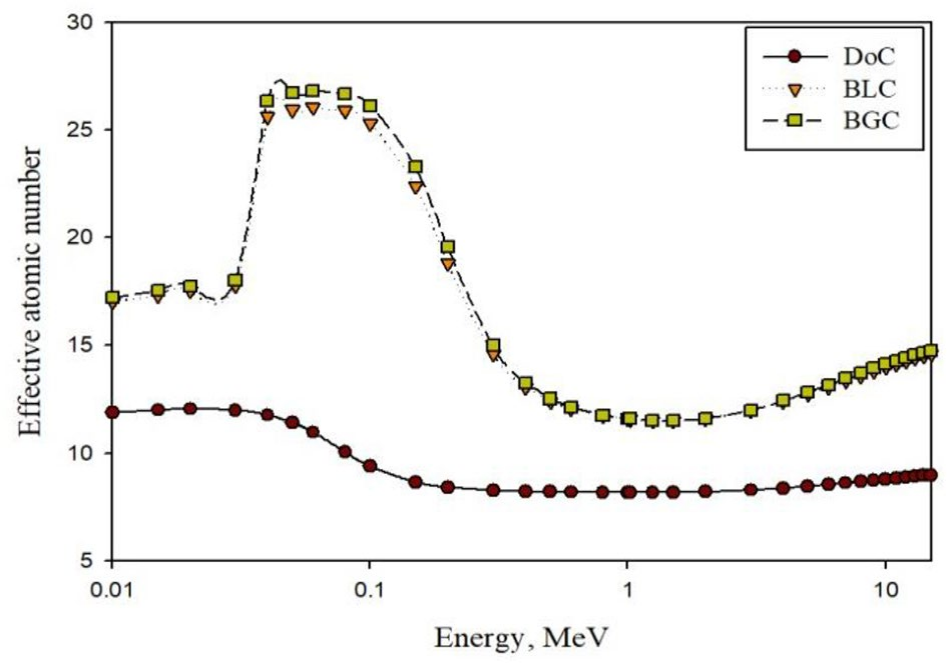
Z_eff_ computed values for the three studied concrete mixes.

First and before discussing the results obtained by the validated MCNP γ-rays and neutronic models, Z_eff,_ which was calculated for the three mixes using the Phy-X program and the macroscopic thermal neutrons absorption cross-section (*Σ*_*abs*_) computed using JANIS-4 software, are presented as shown in Fig. 7 and Table 8, respectively.

**Table 8:** Thermal neutrons attenuation parameters (Σ_Abs_ and λ) were computed using JANIS-4 for the three studied mixes.

Thermal neutrons attenuation parameters					
DoC		BLC		BGC	
*Σ*_*Abs*_ (cm^−1^)	λ, cm	*Σ*_*Abs*_ (cm^−1^)	λ (cm)	*Σ*_*Abs*_ (cm^−1^)	λ (cm)
0.024	42.19	0.057	17.48	5.918	0.169

Based on the obtained results, the effective atomic number for barite mixes, BLC, and BGC, possess significant values higher than that for DoC for the entire studied energy range. The reason can be attributed to their significant contents of high-Z elements such as iron and barium due to employing barite as coarse aggregates and limonite/goethite as fine aggregates^13,46^.

Considering that Z_eff_ is varied with the incident photon energy the same way as the main photon-matter interaction mechanisms do, it can be seen that the barite mixes show the highest values at low energies due to the dominancy of the photoelectric mechanism, whose cross-section directly proportions to nearly Z^4.5^, with slight superiority to BGC over BLC mix which, again, can be attributed to the mix’s content of the heavy elements. After that, at intermediate energies, the differences between the barite mixes and the DoC mix become the smallest as the barite mixes possess the lowest Z_eff_ values at this energy region due to the dominancy of Compton scattering, which is the least Z-dependant mechanism. Finally, at the higher energy range, a regain in the superiority of the barite mixes over the DoC mix can be observed and increases with the photon’s energy as the dominancy migrates gradually to the pair production interaction mechanism, which is in direct proportionality with logEγ and Z^2^^13,46^.

The importance of calculating Z_eff_ is that it helps to assess the shielding efficiency against energetic photons as it indicates the electronic cloud that participates in the photon-atom interaction process and provides early prediction about the attenuation capabilities of the used shield^57^.

**Figure 8:**
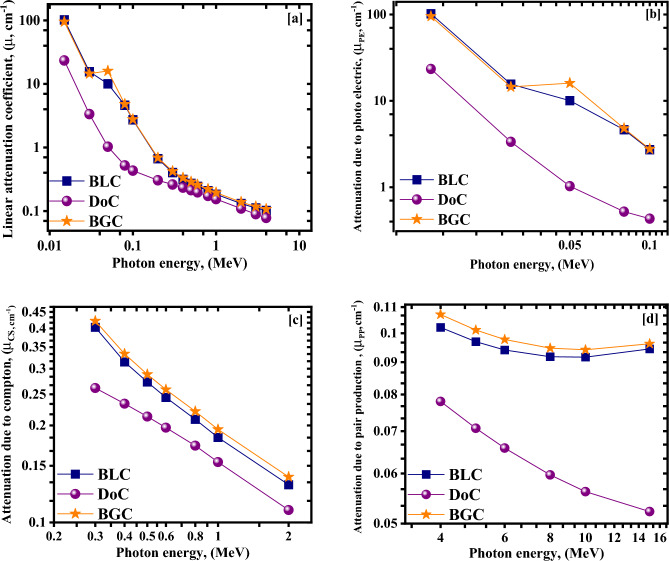
The attenuation coefficients obtained via MCNP5 that represent; (**a**) total linear attenuation, (**b**) attenuation due to photoelectric, (**c**) attenuation due to compton scattering, and (**d**) attenuation due to pair production, focusing on their dominancy regions.

Considering the results obtained by the simulated MCNP γ-rays attenuation model, the linear attenuation coefficients (μ) for the three studied mixes were computed for the energy range starting from 0.015 MeV and ending with 15 MeV. Moreover, a comparison between the investigated mixes based on the three main modes of γ-rays interaction with matter through their prospective dominancy regions has been performed, as shown in Figs. 8a–d.

As predicted earlier based on the obtained Z_eff_ values, barite mixes, BGC and BLC, have shown significant superiority over the dolomite mix, DoC, for the entire studied energy range when observing the obtained linear attenuation coefficients with slight observed superiority of BGC over BLC for the entire studied energy range. Focusing on the dominancy regions of the three modes of interaction, attenuation capabilities of the barite mixes via a photoelectric mechanism at low energies were found to be significantly higher than that for the DoC mix, the same as noticed while computing the effective atomic numbers, which is attributed to the higher content of high-Z elements for barite mixes compared to that contained by the dolomite mix and the significant dependency of the photoelectric mechanism on the atomic number. Again, at intermediate energies, 0.2–2 MeV, the dominancy of Compton scattering is obvious, and the attenuation capabilities between the three mixes become closer due to the weak dependency of the mentioned mechanism on the atomic number. After that, the regain in the former superiority is observed after 4 MeV till the end of the studied energy range due to the significant pair production attenuation; however, the differences are not with the same significance between the barite mixes and the dolomite mix-like those observed at the low energy range as the pair production mechanism depends only on the squared value of the atomic number.

**Figure 9:**
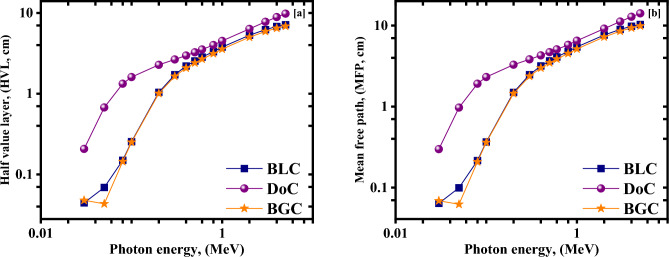
(**a**) The half value layer, and (**b**) mean free path (MFP) versus the photon energy for the investigated concrete mixes.

To reinforce the former interpretation, the mean free path (MFP), which is the average distance that can be traveled through the attenuating medium before the photon can make an interaction, and the required half-value thickness/layer (HVL), which is needed to attenuate 50% of the incoming photons, are computed via the employed MCNP model as shown in Fig. 9a,b.

As expected, the DoC mix shows the greatest thicknesses and, thus, the lowest attenuation capability for the entire energy range, with the most significant differences observed at the low energies. BGC and BLC mixes have very close values, notably smaller than those observable for DoC, especially at low energies.

Returning to the macroscopic thermal neutrons absorption cross-section (*Σ*_*abs*_) values computed using JANIS-4 software, shown in Table 8, the designed BGC mix shows a tremendous improvement regarding absorption of thermalized neutrons with an increased percentage over the DoC mix reaches about 2.5E04% and over the BLC mix reaches 1E04%. The reason for this huge improvement is the addition of the fine powdered boron carbide powder that contains the B-10 element, which is the main responsible for having such a significant (*Σ*_*abs*_) value “5.918 cm^−1^”^25^.

**Figure 10:**
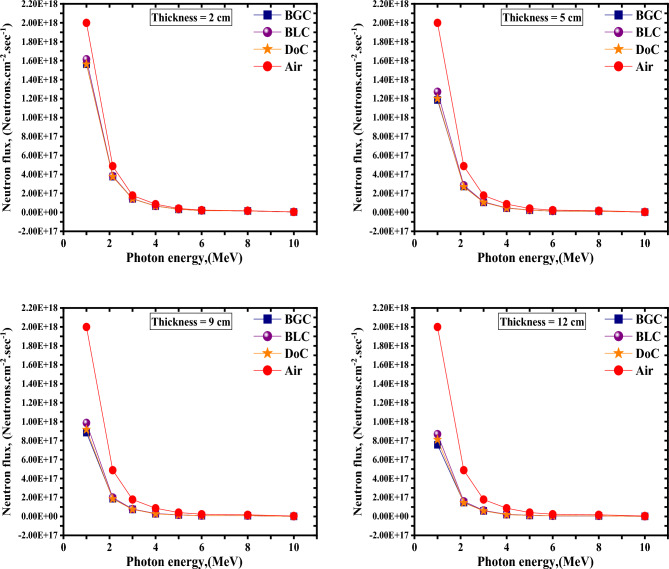
Neutron spectra behind different thicknesses for the studied concrete mixes.

**Figure 11:**
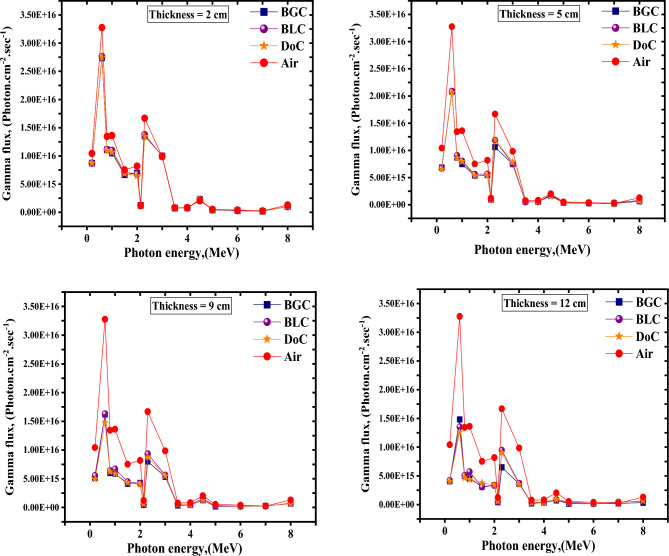
Total γ-rays spectra, primary plus secondary, behind different thicknesses for the studied concrete mixes due to neutrons attenuation.

Considering the simulated MCNP neutrons attenuation model, the obtained results to compare the three studied mixes are presented in Figs. 10 and 11 and Table 9.

**Table 9:** Fast neutrons attenuation parameters (Σ_R_ and λ) were computed using the MCNP5 model for the three studied mixes.

Fast neutrons attenuation parameters					
DoC		BLC		BGC	
*Σ*_*R*_ (cm^−1^)	λ (cm)	*Σ*_*R*_ (cm^−1^)	λ (cm)	*Σ*_*R*_ (cm^−1^)	λ (cm)
0.107	9.35	0.101	9.90	0.108	9.25

Based on the results above, the designed BGC mix was the best in attenuating fast neutrons with slight superiority over the DoC mix, which comes in second place.

The light element content, powdered boron carbide additive, and possessing the highest density are the reasons that have led to putting the BGC mix in the first place regarding shielding against fast neutrons.

Considering the total γ-rays spectra, which are the primary γ-rays emitted from the source plus secondary γ-rays due to any possible nuclear reactions with the shield constituents, observed after the concrete mix varied thickness, it can be seen that the peak observed at about 2.2 MeV, which is attributed mainly to the H(n,γ)D reaction^21,58^, decreases notably with increasing the BGC mix thickness. The cross-section of the former reaction increases notably with decreasing the neutron energy. It becomes significant with thermalized neutrons^54,58^, which is a critical concern when dealing with shielding applications, especially in mixed radiation fields, as it is unacceptable to have a composite shield in such fields that absorbs thermal unenergetic neutrons and, in return, emits hard γ-rays with such high energies.

The reason for the notable gradual suppression of the H(n,γ)D reaction with increasing the thickness of the BGC mix in comparison to the other mixes is that the ^10^B(n,α)^7^Li, due to the mix’s boron content, is considered a strong competitive and the most important is that the gamma photons that may be observed due to this reaction are considered soft with energy equals 0.48 MeV^25,58^ only and that’s why, around this energy, at the maximum studied thickness “12 cm”, BGC mix has shown the highest photons’ flux and in the same time the lowest flux at the 2.2 MeV photon energy in comparison to the other studied mixes.

## Conclusion

Based on the intensive experimental and analytical investigation conducted through this study, the following conclusions were drawn;

1. Specifically, according to the study results, using barite and hydrous goethite as aggregates while considering the addition of fine boron carbide powder can yield a universal radiation shielding concrete that is effective in shielding against energetic photons and poly-energetic neutrons and, at the same time, doesn’t compromise or even enhance the physic-mechanical properties of the final concrete
2. While designing an effective radiation shield, the physical and mechanical properties must be considered along with the radiation shielding properties according to the intended application and use
3. Concrete is an optimal composite shield that gives a chance to use or add different materials simultaneously in the same matrix; thus, it must be effectively designed to contain suitable materials to shield against all kinds of hazardous radiations that may exist and can cause external threats
4. To obtain effective radiation shielding, secondary emitted radiation that may arise from nuclear reactions with the shield constituents, especially during attenuation and absorption of neutrons, must be considered and dealt with
5. Using reliable, validated software programs to investigate a well-designed composite shield can provide reliable estimation for the overall radiation shielding capabilities of the studied shield before production, saving effort, time, and costs.

## Data Availability

All data generated or analyzed during this study are included in this published article.
